# Impact of Austria's 2009 trans fatty acids regulation on all-cause, cardiovascular and coronary heart disease mortality

**DOI:** 10.1093/eurpub/cky147

**Published:** 2018-10-29

**Authors:** Igor Grabovac, Lisa Hochfellner, Matthias Rieger, Jo Jewell, Andrew Snell, Adelheid Weber, Hans-Peter Stüger, Karin E Schindler, Bente Mikkelsen, Thomas E Dorner

**Affiliations:** 1 Department of Social and Preventive Medicine, Centre for Public Health, Medical University of Vienna, Vienna, Austria; 2 Department of Statistics and Analytical Epidemiology, Austrian Agency for Health and Food Safety, Graz, Austria; 3 International Institute of Social Studies of Erasmus University Rotterdam, The Hague, The Netherlands; 4 World Health Organisation Regional Office for Europe, Copenhagen, Denmark; 5 Department of Maternal, Child and Gender Health and Nutrition, Federal Ministry of Labour, Social Affairs, Health and Consumer Protection, Vienna, Austria

## Abstract

**Background:**

Unhealthy diet, especially consumption of trans fatty acids (TFAs), is a known risk factor for cardiovascular disease (CVD), a leading cause of death in Austria. In 2009, Austria introduced a law regulating the content of TFAs in foods. The aim of this study was to assess the impact of the TFA regulation on CVD-related outcomes.

**Methods:**

The study evaluated the TFA regulation as an intervention in a natural experiment. Two study periods were assessed: pre-intervention (1995–2009) and post-intervention (2010–14). The study compared the age-standardized death rates per 100 000 population for CVD outcomes with those of a ‘synthetic’ international comparator population, created from data of OECD countries where TFA regulation has not been implemented, but where the population is otherwise comparable.

**Results:**

There was a continuous decrease in CVD-related mortality throughout the study period in both the synthetic international comparator population, as well as in the adult Austrian population, with no significant change in this trend observed as an effect of TFA regulation.

**Conclusions:**

Whilst the results are counterintuitive, given the established link between TFA consumption and an increased risk of CVD, there are many possible explanations: high prevalence of tobacco smoking, changes in TFA content in foods due to international guidance as opposed to formal regulation and a beneficial impact of TFA regulation on sub-groups of the population that might not be detected with nationally aggregated data. However, reduction in TFAs should still be considered an important part of risk factor reduction for CVD and other non-communicable diseases.

## Introduction

A growing number of health problems [such as cardiovascular diseases (CVD), obesity, hypertension, stroke, type II diabetes mellitus, chronic kidney disease, cognitive insufficiency, atrial fibrillation] are caused or influenced by dietary patterns through adverse effects on glucose-insulin homeostasis, oxidative stress, inflammation, endothelial health, hepatic function, adipocyte metabolism, visceral adiposity etc.^1^^,^^2^ The cardio-metabolic consequences of different dietary patterns and nutrient intakes have been researched to varying degrees, using a variety of methods, including interventional trials, prospective cohorts and randomized controlled trials.^2^ This has led the World Health Organization (WHO) and national authorities to adopt nutrient- and food-based dietary guidelines, including the publication of updated WHO guidelines on sodium intake, free sugars and, most recently, saturated and trans fatty acid (TFA) fat intake in 2018.^3^ The promotion of healthy diets is now a global policy priority for public health, as reflected in increasing commitments by countries at national and international levels.

TFAs are unsaturated fatty acids that contain at least one non-conjugated double bond in the trans configuration and can be found naturally in ruminant foods (rTFA) or in industrial produced oils (iTFA). iTFAs were widely used during the 1970s–80s when margarine was advocated as a butter substitute due to the lower saturated fat content.^4^ However since the 1990s it has become evident that high intake of TFA is associated with many negative health outcomes.^5^ Based on multiple reports, it was concluded that there were no nutritional benefits of TFA and there were clear adverse effects especially in increasing the risk and mortality of CVD, therefore limiting their consumption and contents in various food items should be a policy priority.^6^ Based on the available evidence, reviewed according the GRADE methodology, the WHO has recommended reducing the intake of TFA to <1% of total energy intake among children and adolescents.^3^ Denmark was the first country globally to introduce a trans-fat ban via legislation adopted in 2003, setting limits for industrially produced TFA in the food supply (<2 g TFA per 100 g total fat).^7^ Many countries have since followed the Danish example and adopted policies to reduce or eliminate of TFAs in food products.^8^

In Austria, a regulation of TFA in foods was introduced in 2009.^9^ Prior to this, monitoring of the food supply demonstrated that food products available on the Austrian market contained very high levels of TFA. This was particularly pronounced in margarines, convenience food products and fast foods, which contained on average 7.83, 3.64 and 2.17% TFA/FA, respectively. In fact, before 2008, 42% of the sampled food products (including various convenience food products, fast food as well as margarines, oils and cooking and frying fats) had a TFA content over 2% and more than 10% of the products had a TFA content over 10% of total energy.^10^ The food regulation law regarding the content of TFA in food set limits on the use of TFAs to 2/100g of total fat content. For multi-ingredient processed foods with a total fat content below 20%, TFAs were restricted to 4/100g of total fat and for processed foods with a total fat content below 3% TFA to 10/100 g of total fat.^9^

We aimed to investigate if these regulations led to a significant reduction in cardiovascular outcomes such as CVD and mortality due to coronary heart disease (CHD) in Austria. As CVD (ICD-10 diagnoses I00-I99) and CHD (ICD-10 diagnoses I20-I25) specific mortality are multimodal and influenced by a variety of individual risk factors such as age, smoking habits, hypertension, GDP and healthcare provision,^8^^,^^11–13^ these factors must be taken into consideration during the analysis.

## Methods

### Data extraction and sources

Data were extracted from the OECD database and are comparable across OECD members.^14^ Our study period was between 1995 and 2014. A pool of comparator countries were chosen if they did not implement a TFA regulation during the study period and if data points were available for more than 60% of cases in the database. Our original data set included 13 countries: Australia, Estonia, Finland, France, Ireland, Israel, Italy, Luxembourg, Mexico, New Zealand, Portugal, Sweden and UK) with data available from 1995 until 2014. If date for single years in the respective countries were missing, the values were replaced using linear interpolation.

### Intervention and dose

The introduction of the TFA regulation in the year 2009 is a public health intervention enacted by the Ministry of Health, Families and Youth (currently Ministry of Labour, Social Affairs, Health and Consumer Protection) in response to an analysis of the TFA content of food (in g TFA/100g total fat). These data are provided in table 1. Our study has two study periods: before (1995–2009) and after (2010–14) the TFA regulation.

**Table 1:** Trans fatty acid (TFA) content in food samples (g TFA/g fat) 2007–09 (before) vs. 2010–17 (after) introducing TFA regulation in Austria

	Before TFA regulation			After TFA regulation			
	Number of samples	Mean	Median	Number of samples	Mean	Median	*P*-value
All	535	3.21	0.60	2912	0.70	0.29	<0.01
WG-0501	132	8.22	1.00	250	1.31	0.48	<0.01
Vegetable fats, margarine							
WG-0502	12	5.29	0.90	1163	0.22	0.11	<0.01
Vegetable oils							
WG-0702	218	1.89	0.49	501	1.13	0.40	0.01
Fine bakery products—baked by confectioner							

### Outcomes

The main outcomes of interest were all-cause mortality, CVD mortality (mortality due to ICD-10 diagnoses I00-I99) and CHD mortality rates (mortality due to ICD-10 diagnoses I20-I25) per 100 000 people annually.^15^ The following potential confounders were taken into account: age and gender distribution of the population, GDP per capita, health expenditures per capita as well as the prevalence of smoking, obesity, hypertension and diabetes mellitus.

### Statistical analysis

This study quantifies the effect of the Austrian TFA regulation exploiting a natural experiment,^18–20^ comprising of two elements: (i) the change of outcomes before and after the introduction of the regulation; (ii) comparator countries which did not have any such TFA related policies.^16–18^ We employed the ‘synthetic control method’ and identified comparator countries according to data trends similar to that of Austria before the intervention. If the difference in pre-intervention trends between Austria and its ‘synthetic control’ are sufficiently small, corresponding post-treatment differences may be plausibly attributed to the effect of the regulation.^19^ The underlying assumption is that unobserved confounders remain unchanged following the intervention.

Our empirical model compared Austria’s reported all-cause mortality, CVD mortality and CHD mortality rates over time with those of the synthetic control group. The synthetic control group was composed as a weighted average of the OECD countries included in the analysis.^20^ The weights assigned to the control countries were non-negative and equal to the sum of one. A recursive algorithm selected the weights optimally so that the synthetic control group most closely resembled the mortality rates and risk factors in Austria in the pre-treatment period.

Data preparation and all statistical analysis were carried out by using the software GNU R version 3.4.4.^21^ The model was calculated using the synthetic control method, with all statistical routines being implemented using the R package MSCMT.^22–24^

The synthetic control method does not lend itself to classical hypothesis testing given that there is only one treatment unit and no sampling error due to the use of aggregate data. Instead, inference is exact and the test assesses whether effect sizes ‘stand’ out from the data. We conducted a series of placebo tests by iteratively and falsely applying the TFA regulation in 2009 to each of the 13 synthetic control countries (shifting Austria to the control pool). Afterwards, we computed the estimated effect associated with each placebo run. This iterative procedure gives a distribution of estimated gaps for the control countries where no TFA regulation had been introduced. With the results of the placebo tests, pseudo-*P*-values were computed by calculating and ranking the ratios of post- to pre-intervention root mean squared prediction errors for all Austria and all placebos runs.

Austria did not fully comply with the WHO Framework Convention on Tobacco Control, especially in the implementation of ‘Protecting people from tobacco smoke’ (banning smoking in public places) by introducing exceptions (such as designated smoking rooms, or allowing for smoking and non-smoking places). As tobacco is a major risk factor and potential confounder, we used sensitivity analysis to assess its influence: we excluded those OECD countries with full implementation of the ban in public places—Australia, Ireland, New Zealand and UK. The final list of nine included countries was Estonia, Finland, France, Israel, Italy, Luxembourg, Mexico, Portugal and Sweden.^25^

All calculations were done separately for women, men and both sexes together.

## Results

In table 1, the TFA contents of food samples in Austria are shown. There was a remarkable and significant decrease of TFA contents in total, as well as in vegetable fats, margarines, vegetable oils and fine bakery products following the introduction of TFA regulation in Austria.

**Figure 1:**
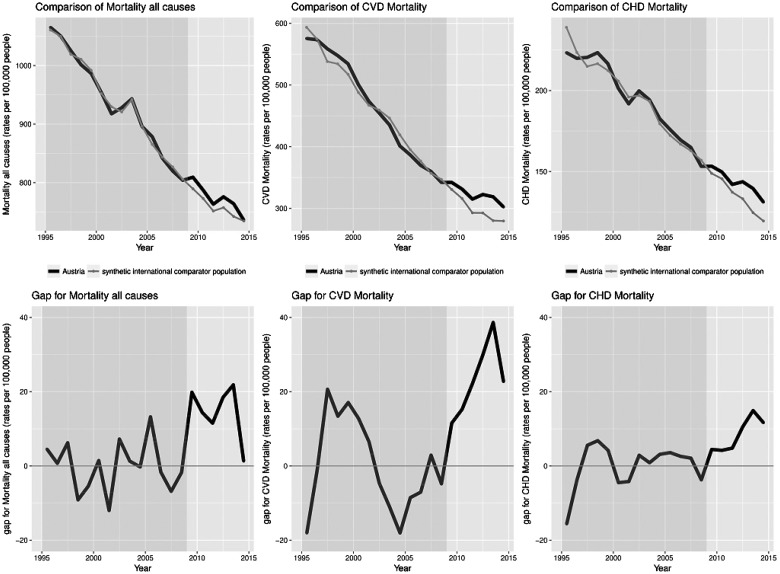
Comparison of all-cause, CVD and CHD mortality rates per 100 000 people in both sexes with yearly gaps in mortality between Austria and synthetic international comparator population

A comparison of Austrian data for all-cause mortality as well as CVD and CHD specific mortality and synthesized data for synthetic Austria is shown in figure 1, where both the comparison as well as plotted gaps (i.e. differences between original and synthesized data) may be seen. The all-cause, CVD and CHD mortality in synthetic Austria tracks the trajectories of the variables closely for the entire period before the TFA regulation. Therefore, the synthetic international comparator population provides a sensible counterfactual of all-cause mortality during 2010–14 in the absence of the TFA regulation. The year-per-year effect of the TFA regulation on mortality is the trend difference. Following TFA regulation the two trends began to diverge slightly. While the mortality in synthetic international comparator population continued its moderate downward trend, Austria experienced a small increase. These empirical patterns do not point to any reductive effect of the TFA regulation on all-cause mortality.

### Sex differences

**Figure 2:**
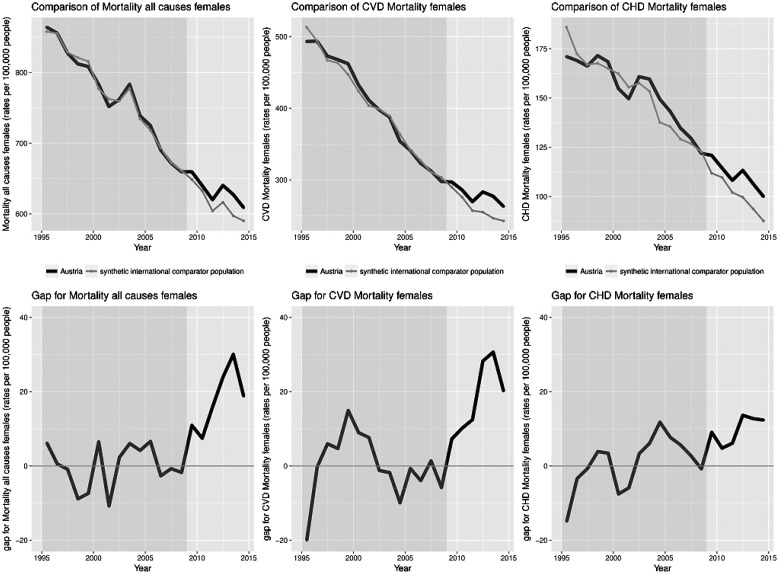
Comparison of all-cause, CVD and CHD mortality for women rates per 100 000 with yearly gaps in mortality between Austria and synthetic international comparator population

**Figure 3:**
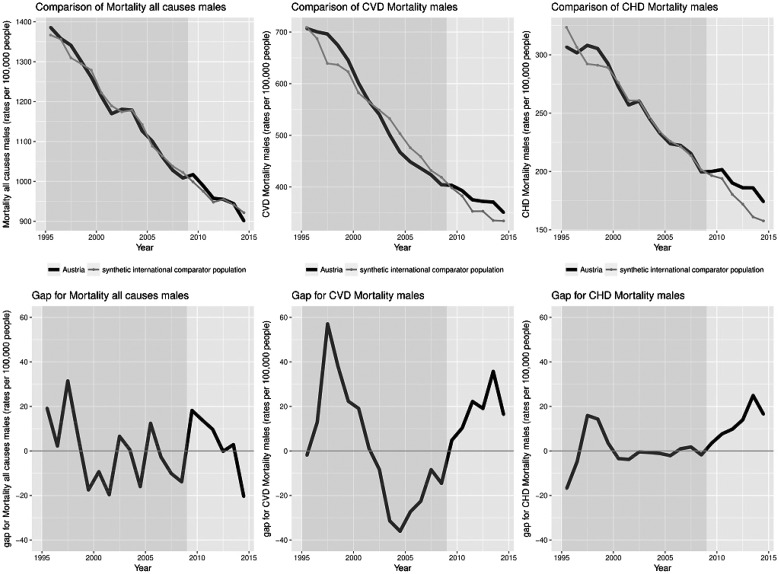
Comparison of all-cause, CVD and CHD mortality for men rates per 100 000 with yearly gaps in mortality between Austria and synthetic international comparator population

Data stratified by sex shows similar trends, presented in figures 2 and 3. The gap between Austria and synthetic Austria in the trends of mortality after introduction of TFA regulations was slightly more pronounced in women compared to men.

### Placebo tests

**Figure 4:**
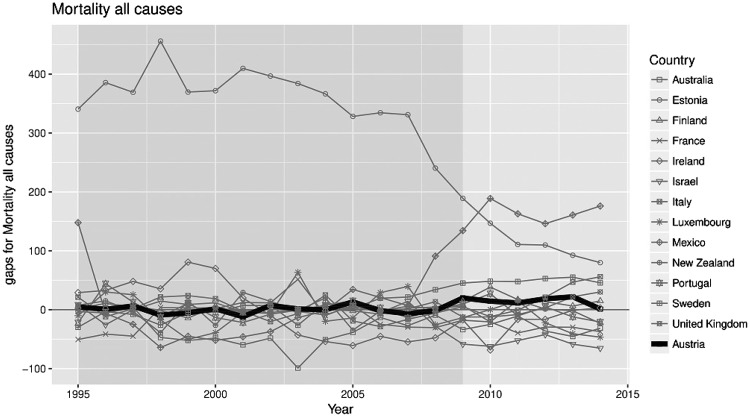
Placebo plot: all-cause mortality gaps in Austria and placebo gaps for all 13 synthetic control countries

Figure 4 illustrates results of the 13 placebo tests. The dashed lines represent the gap between a given country and its synthetic counterpart. The continuous line highlights the gap estimated for Austria (corresponding to figure 1b). As shown in figure 2, the placebo tests created gaps of magnitude similar to those estimated for Austria. Therefore the analysis did not provide significant evidence of a (negative or positive) effect of the TFA regulation in Austria. Additionally, figure 4 indicates that all-cause mortality trends during the period 1995–2009 cannot be reproduced well for some countries by a weighted average of control countries. Nevertheless excluding control countries with large pre-treatment errors such as Estonia did not affect the results. All pseudo-*P*-values were above the significance level of 0.05. The series of placebo studies did not confirm any (pseudo-)significance of the estimated effect.

In unreported analysis, the synthetic control method and the placebo tests were applied to CVD and CHD mortality for women, men and both sexes. The results were qualitatively similar across outcomes and groups, suggesting no discernible effect of the regulation. Performing a sensitivity analysis involving the exclusion from the synthetic control of countries that introduced a smoking ban regulation during the study period did not change these findings.

## Discussion

This analysis evaluated the Austrian policy ban on TFA in the food supply. The paper is the first to show the effects of an implementation of such a policy in Austria on a population level. As shown in table 1, the reduction of the TFA concentration in vegetable oils, margarine and fine bakery products showed a significant reduction following the TFA regulation in an analysis of food products. Given numerous studies that confirm the increased risk of CVD and CHD when consuming TFA,^3^^,^^6^ and due to an actual reduction occurring, we expected to observe a decrease in our outcome variables, the all-cause, CVD and CHD mortality. However, even though the synthetic international comparator population trends follow the observed Austria trends prior to the policy, providing a very good approximation, we were unable to demonstrate a decrease in the mortality trends associated with the TFA policy. Therefore, our results contradict similar research from other countries as well as observational studies. For example, a 2016 Danish study reported a rapid decrease in CVD mortality rates after the implementation of a similar TFA policy, reporting a decrease in CVD mortality rates of about 14.2 death per 100 000 people per year relative to the synthetic control.^19^ Following a policy on TFA reduction in gastronomic establishments in New York, it was found that there was a 4.5% reduction in CVD mortality. The authors further reported that this reduction could be valued at 3.9 million dollars US annually per 100 000 people.^26^

Our results note an overall decrease in all-cause, CVD and CHD mortality over the investigated period (figures 1–3), however, paradoxically the mortality trends have declined even further in the synthetic international comparator population following the TFA policy implementation, which suggests that the policy implementation alone has no lowering effect on all-cause, CVD and CHD mortality. These differences may be driven by a number of alternative influences on diet and CVD outcomes.

There were differences in the trends of tobacco smoking prevalence, a known risk factor for all-cause as well as CVD and CHD mortality. Countries that were used in the calculations for synthetic Austria show an overall decline in smoking prevalence.^25^ Unfortunately, this is not the case for Austria, where smoking prevalence trends do not show a decrease, with the prevalence in men reported as showing a stable trend and a significant increase in smoking prevalence in women.^27^ This could further contribute to the explanation of our results stratified by sex (figures 2 and 3), where the gap between Austria and synthetic Austria was more pronounced in women. The sensitivity analysis and exclusion of countries was based on the countries that have implemented a smoking ban in public places. However, there are many other components to effective tobacco control, including taxation, which were not considered and might have varied between Austria and synthetic international comparator population.

A beneficial effect on sub-groups in Austria might not have been detected in the aggregated data used here. A study using UK and Dutch data showed that women and non-native population tend to be among the top consumers of TFAs and so possibly more sensitive to regulation, while socioeconomic background had no effect.^28^

Furthermore, a ‘spill-over’ effect of TFA might have played a part in two different ways: TFA regulation and a growing awareness of the harms of TFA might have had an international effect reducing TFA use in the synthetic international comparator populations; high-TFA foods may continue to be available in Austria through importation. A study on TFA contents in food products in Portugal reported that readily available, cheap and highly consumed pastry products remain an important source of TFA. Especially worrying were imported pastry products found in ‘low cost shops’.^29^ Although such imports may have contributed to our null finding, this should not be a major influence as the analysis of the TFA content in Austrian food products has shown a drastic decrease (table 1).

Moreover, matching a small set of countries to Austria (which differ along many dimensions) using synthetic international comparator population data remains a relatively crude exercise, which may explain the null finding. Another potential limitation is that the introduction of a specific regulation does not constitute a clear end, but rather that there is always a ‘wash in’ period in which the industry has to comply with the regulation followed by the ‘wash out’ period after the full implementation of the regulation but in which the industry has still not fully complied. We were not able to clearly differentiate these periods in the analysis. Additionally, there is a possibility that the lack of effect is due to the analysis chosen, which might have been different if multiverse analysis (calculating analyses across a whole set of alternatively processed data sets corresponding to a large set of reasonable scenarios) approach was used.^30^ However, in this paper we opted for the ‘synthetic control’ method, and have tried various different analyses (age stratification, with and without inclusion of smoking regulation or health expenditure and GDP) yet none of these variations generated a different result.^22^

In conclusion, whilst the findings of this study are in some ways counterintuitive, knowing the link between TFA consumption and an increased risk of CVD and CHD, there are many possible alternative explanations that this study was not able to test. Reduction in TFAs should still be considered an important part of risk reduction for CVD, CHD and other non-communicable diseases.

This study contributes to the pragmatic research to evaluate public health interventions, and the use of natural experiment studies as part of this. However, to enable more in-depth and meaningful evaluations, countries and the international community should continue to strengthen monitoring and data collection, and efforts should be made to further increase the capacity for high quality evaluations that can influence national policies and implementation of major risk factor control.
